# Biomechanical Behavior of an Aged Synthetic Hernia Mesh Under a Cough-Simulating Protocol

**DOI:** 10.3390/ma19163368

**Published:** 2026-08-07

**Authors:** Miglena G. Kirilova-Doneva, Dessislava L. Pashkouleva

**Affiliations:** 1 Faculty of Pharmacy, Medical University of Sofia, 1000 Sofia, Bulgaria; 2 Institute of Mechanics, Bulgarian Academy of Sciences, 1113 Sofia, Bulgaria; dessip@imbm.bas.bg

**Keywords:** hernia mesh, uniaxial cyclic tensile tests, mechanical properties, cough simulation

## Abstract

Hernia meshes are repeatedly subjected to physiological loading caused by fluctuations in intra-abdominal pressure during daily activities. This study aimed to investigate the effects of prolonged cyclic loading simulating repetitive severe coughing on the mechanical properties of expired, non-implanted Microval polypropylene mesh. Uniaxial cyclic tensile tests were performed on aged Microval mesh specimens in the two principal material directions. The loading protocol simulated repetitive coughing by applying six loading sessions per day over three consecutive days. Residual strain, elastic modulus, and loading duration were evaluated throughout the experiment. After the loading protocol, the specimens were tested to failure. Cyclic loading affected the mesh. Strain was within 12–20% in the longitudinal (L) direction and up to 25% in the transverse (T) direction after the third testing day. The elastic modulus increased significantly with repetitive loading, indicating pronounced material stiffening, while the ultimate tensile strength remained within the range measured for the unused specimens. In addition, the first loading cycle was approximately 20% longer than the subsequent cycles. Prolonged cyclic loading caused significant permanent deformation and pronounced material stiffening in the aged Microval mesh, whereas its ultimate tensile strength was only slightly affected. These findings enhance understanding of the mechanical behavior of hernia meshes under repetitive physiological loading.

## 1. Introduction

Hernia meshes are investigated through both in vivo and in vitro experiments. In vivo experiments are performed after mesh implantation in patients, and their mechanical response can be assessed using non-invasive techniques such as optical methods for deformation analysis, ultrasound shear wave imaging combined with electromyography, and/or intra-abdominal pressure measurements [1,2]. In vivo studies also include the assessment of mesh biocompatibility and long-term performance using imaging techniques such as magnetic resonance imaging (MRI), as well as histological analysis. These approaches enable the investigation of tissue integration, inflammatory reactions, and possible complications, such as adhesions or hernia recurrence. The information obtained contributes to a better understanding of the behavior of hernia meshes within the human body.

In the case of in vitro experiments, both static and dynamic tests are applied. According to Whitehead-Clarke et al., authors usually use static experiments and animal models [3]. Actually, dynamic tests are those that reveal the main characteristics of the meshes. Physiological activities such as sitting, breathing, coughing, and jumping, during which the abdominal wall is subjected to elevated intra-abdominal pressure (IAP), can be modeled by dynamic tests. The main question during modeling these processes is how to choose the initial conditions (the values of IAP and force applied to the abdominal wall) as well as the physiological limits of the parameters.

In 1973, Kirsch measured that intra-abdominal pressure varies from 0.2 kPa at rest to maximum values reaching approximately 20 kPa, corresponding to about 150 mmHg [4]. The maximum values of intra-abdominal pressure in humans, measured by Cresswell et al., are in the range of 11–15 kPa [5]. Later, Cobb et al. reported that average IAP is in the range of 80–90 mmHg (approximately 10.7–12.0 kPa), but increased values are up to 150–180 mmHg (approximately 20–24 kPa) during coughing and jumping [6]. The results were obtained using a transurethral bladder (Foley) catheter.

Brown et al. estimated that intra-abdominal pressures during coughing and jumping may reach approximately 170 mmHg (about 23 kPa) [7]. These findings suggest that meshes used for hernia repair should be capable of withstanding pressures of at least 180 mmHg under extreme physiological conditions [7].

More recent studies have reported peak intra-abdominal pressures of up to 300 mmHg during coughing measured by a balloon catheter coupled with a pressure transducer system [8]. In another study, peak intra-abdominal pressures of 214 mmHg and 165 mmHg were reported for healthy males and females, respectively [9].

Assuming the abdominal cavity to be a thin hollow sphere, Klinge calculated the maximum load exerted on the abdominal wall using Laplace’s law and stated that if the longitudinal diameter of the abdominal cavity is 32 cm, this results in a load of 16 N/cm along the circumference of the cavity [10]. In the transverse direction, with an abdominal diameter of 8 cm, the load is 4 N/cm [10]. Using the same law, Cobb calculated that for an abdominal circumference of 100 cm, the load exerted on the abdominal wall is 32 N/cm at an intra-abdominal pressure of 170 mmHg [6]. Klosterhalfen et al. calculated the same load—32 N/cm for the same abdominal circumference of 100 cm and an IAP of 20 kPa (150 mmHg), with the thickness of the abdominal wall not specified [11].

Deeken et al. calculated tensile stresses in the abdominal wall using both spherical and cylindrical models of the abdominal cavity subjected to internal pressure. Parameters such as abdominal circumference, gender, body mass index, and IAP were included in the analysis [12]. Their results showed that the theoretical stresses were higher for the cylindrical model (21–47.8 N/cm) than for the spherical model (10.5–23.9 N/cm). Based on these findings, Deeken et al. concluded that extreme loads reach 42–47 N/cm [12].

It is generally accepted that the normal force applied to the abdominal wall is about 16 N/cm and that the meshes need to withstand a maximum load of about 32 N/cm, which is considered representative under physiological extreme conditions [6,10,11].

Several studies have reported the behavior of hernia meshes under cyclic loading. These investigations revealed characteristics such as bilinear mechanical behavior, stiffening, and permanent elongation of the mesh specimens [13,14,15,16].

Jones et al. compared the biomechanical properties of five commonly used prolapse meshes (Gynecare PS™ (Ethicon), Pelvitex™ (Bard), Popmesh™ (Caldera), Ti mesh™ (pfm medical GmbH), and Polyform (Boston Scientific)) loaded to failure or cyclically loaded [13]. With the exception of Popmesh™, which displayed linear behavior, all prolapse meshes were characterized by a very low initial stiffness that increased into the high-stiffness region. Pelvitex™, Popmesh™, Ti mesh™ and Polyform™ are significantly less stiff than Gynecare PS™. The significant amount of permanent elongation of these meshes is also discussed. Gynecare PS™ samples remained elongated to 20% of their initial length, which is significantly lower than all other meshes.

Rynkevic et al. studied the mechanical performance of Ultrapro^®^, Dynamesh^®^, and Surgipro^®^ meshes under cyclic loading [15]. The observed deformation is between 10 and 25%. Three meshes underwent permanent plastic deformation, which induced decreased mesh flexibility over time.

A significant non-recoverable extension, ranging from 8.5% to 19%, was also reported by Krause et al. for nine commercially available meshes (Gynemesh^®^, tension-free vaginal tape (TVT), Prolene^®^, suprapubic arc (SPARC), Vypro^®^, Dexon^®^, Vypro II^®^, Atrium^®^, and intravaginal sling (IVS)) [17].

Patterson et al. investigated the effect of cyclic loading on the mechanical properties of Bard Soft^®^, Gynemesh^®^, Marlex^®^, and Mersilene^®^ meshes [14]. Their results demonstrate that the polypropylene meshes experience an increase in stiffness during cyclic loading. The same trend was observed in the study of Li et al. [18] and Tomaszewska et al. [19].

Li et al. characterized the mechanical properties of polypropylene mesh Prolene^®^ using a cyclic test under simulated physiological loading conditions [18]. They observed an increase in stiffness (E modulus increased from 1.09 to 26.5 MPa) and significant non-recoverable extension up to 60%.

Tomaszewska et al. discussed the mechanics of a mesh implanted in the abdominal wall under repetitive loading using experimental and numerical studies [19]. Their results show that the elastic modulus of the mesh approximately doubles under repetitive loading within the physiological strain range.

The present study was designed to assess the response of an aged hernia mesh to repetitive loading, assuming that some time after hernia operation (in our case 3 years), patients develop pulmonary diseases associated with severe chronic coughing, such as chronic obstructive pulmonary disease (COPD), asthma, or idiopathic pulmonary fibrosis (IPF).

The study aims to investigate and discuss the effects of prolonged cyclic loading on the mechanical behavior of expired, non-implanted polypropylene mesh Microval using an in vitro model simulating repetitive intra-abdominal pressure years after the operation.

## 2. Materials and Methods

### 2.1. Specimen Preparation

The specimens were cut from a single piece of Microval mesh whose expiration date had passed 3 years before testing.

As established in our previous work [20], the implant material exhibits orthotropic mechanical behavior, and specimens were cut along two orthogonal directions. The dimensions of the specimens were 70 × 10 mm. This size allows a clamp-to-clamp distance of 40 mm. The specimen width was 10 mm. Three specimens were tested in each material direction. The tests were performed using a modernized FU1000e universal testing machine (FRITZ HECKER, Leipzig, Germany). The crosshead speed was set to 0.5 mm/s, corresponding to an initial strain rate of 0.0125 s^−1^ at the beginning of the test.

The direction of highest material strength was denoted as “L” (longitudinal direction), and the perpendicular direction was denoted as “T” (transverse direction).

### 2.2. Cyclic Loading Protocol

Cyclic loading tests were performed to investigate the mechanical behavior of hernia meshes under physiologically relevant conditions. The loading protocol was designed to simulate repetitive loading associated with coughing.

Coughing is known to raise the intra-abdominal pressure within one second from values below 20 to above 140 mmHg (approximately 2.7–19 kPa). Cobb et al. reported maximum cough pressures of 127 mmHg (approximately 16 kPa) in the sitting position and 141 mmHg (approximately 19 kPa) in the standing position [6].

The specimens were subjected to cyclic uniaxial tensile loading with a force of 11 N/cm. The applied load is constant, regardless of the duration of the cycle episodes, ranging from 0 N to a maximum of 11 N. This load level was selected based on the work of Cobb et al., who reported that the mean intra-abdominal pressure generated during coughing ranges between 40 and 127 mmHg (approximately 5–19 kPa) [6]. It can be estimated that the maximum tensile strength ranges from 4 to 13 N/cm. The chosen loading level also facilitates comparison of the present results with those reported for other commercially available hernia meshes.

The frequency and intensity of coughing episodes were defined according to Birring et al., who investigated hour-to-hour variability in cough frequency in patients with chronic cough [21]. Based on these data, the loading protocol was designed to simulate between 13 and 71 cough events per hour, corresponding to repeated pressure peaks of approximately 11 kPa (80 mmHg) acting on the implanted mesh.

Six cough episodes were simulated each day between 09:00 and 14:00 throughout the three-day experimental period. The specimens were exposed to a variable number of simulated coughs during each hour. Three specimens taken from each direction were tested. During each testing day, the specimens underwent a total of 239 loading cycles. A recovery period of 19 h was provided between daily testing sessions. In addition, a minimum rest interval of 25 min was maintained between consecutive loading episodes to simulate the recovery period between coughing bouts. The cough-frequency ranges considered in the experimental protocol are summarized in Table 1.

The following parameters were recorded: the time required to complete each loading episode in both the L and T directions, as well as the elongation of each specimen at the beginning and at the end of every loading cycle. Because permanent deformation accumulated during cyclic loading, the gauge length was premeasured after each loading episode and used as the reference length for the subsequent loading episode. All strain values were calculated with respect to the updated gauge length, thereby accounting for the progressive accumulation of residual deformation throughout the experiment. The elastic modulus of the material in its initial state and after cyclical loading in each direction was determined using linear approximation. The elastic modulus *E* was defined as the minimum slope over the first 10% interval of the stress–strain curve.

After completion of the cyclic loading protocol, the specimens were subjected to uniaxial tensile testing to failure. The failure load (N) and the corresponding relative elongation at failure after cyclic loading at the end of the daily test were recorded. The strain was calculated with respect to the initial gauge length. The difference between strain at the beginning and at the end of each loading cycle in the L and T directions was calculated.

The chosen parameters were used to evaluate the effect of repetitive loading on the mechanical response of the mesh in both principal material directions.

### 2.3. Statistics

The statistical tests were performed using a statistical software package, MedCalc ver. 17.9.7 (MedCalc Software Ltd., Ostend, Belgium). Quantitative data are presented as mean ± standard deviation. An unpaired Student’s *t*-test was used (two-tailed, confidence level of 95%) to compare the mean values of parameters in different directions where the samples are independent. The level of significance was set to *p* < 0.05.

## 3. Results

**Figure 1:**
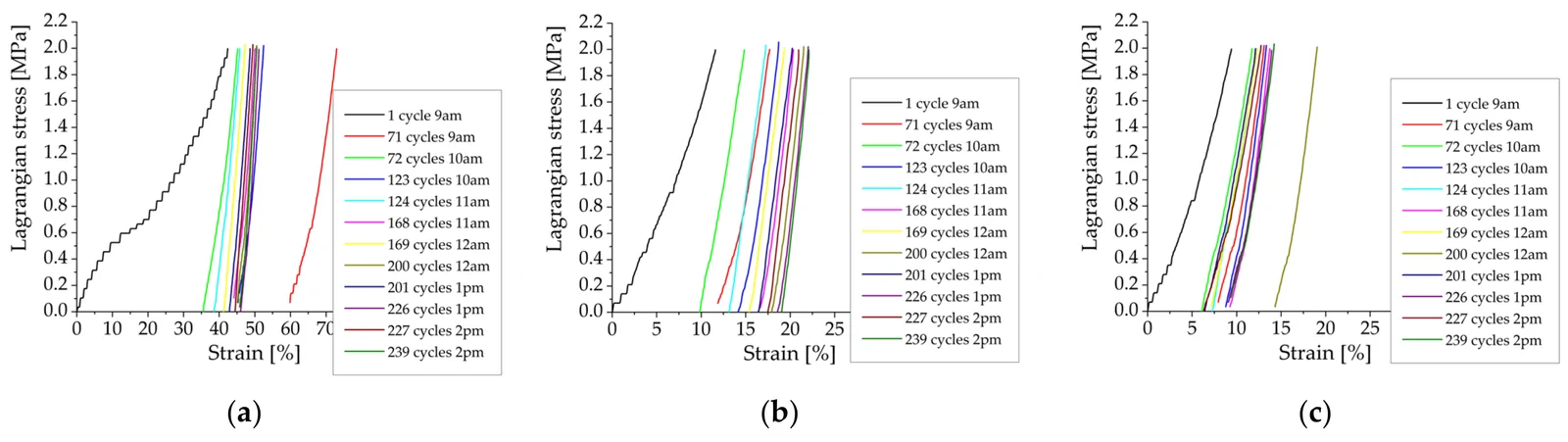
Lagrangian stress–strain curves of Specimen 1 in the L direction after (**a**) the first, (**b**) the second, and (**c**) the third day of repetitive loading.

**Figure 2:**
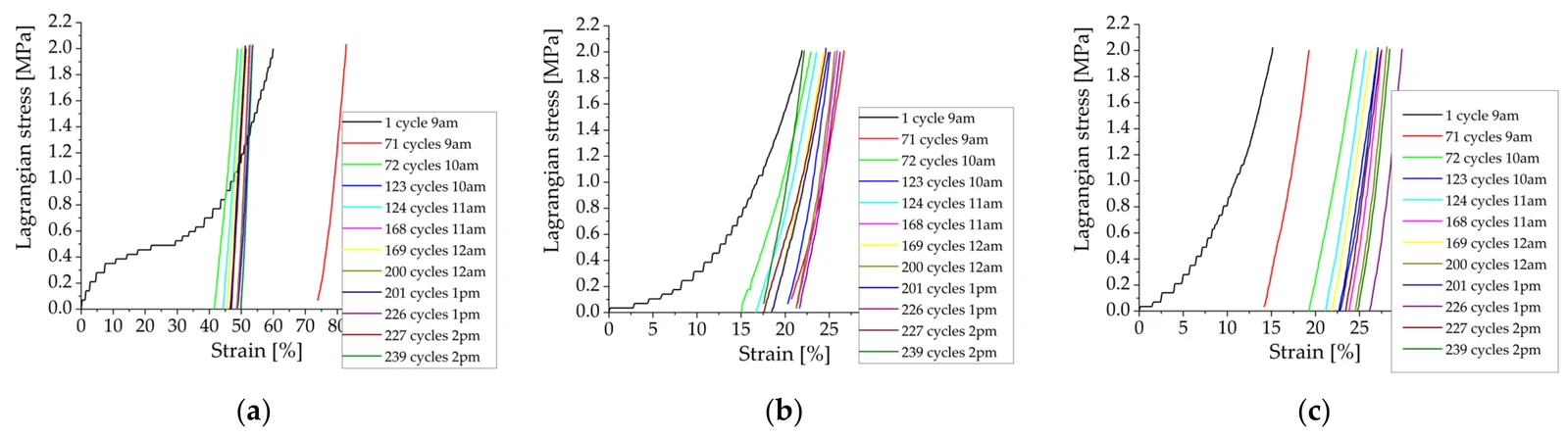
Lagrangian stress–strain curves of Specimen 1 in the T direction after (**a**) the first, (**b**) the second, and (**c**) the third day of repetitive loading.

The influence of cyclic loading on the mechanical properties of hernia mesh Microval was investigated. All specimens were subjected to cyclic loading at a force of 11 N. The number of cycles was determined based on the hour-to-hour variability in cough frequency during a monitoring period reported by Birring et al. [21] (see Table 1). The experimental load–elongation data were converted into stress–strain curves for subsequent analysis and comparison. The stress–strain responses of one specimen (S1) loaded in the L and T directions after the loading scenario are presented in Figure 1 and Figure 2, respectively. The stress–strain curves obtained in both directions exhibited a characteristic bilinear behavior, consisting of two distinct deformation regions. This bilinear response was more pronounced in the T direction than in the L direction. In both directions, the initial deformation stage was characterized by a low-stiffness region, which gradually transitioned into a second region exhibiting substantially higher stiffness (Figure 1 and Figure 2). For quantitative analysis, the low-stiffness region was defined by the minimum slope calculated over the initial 10% strain interval. The high-stiffness region was defined by the maximum slope calculated over a 30% strain interval.

The increase in gauge length resulting from cyclic loading between 0.2 and 11 N (13–71 cycles per loading episode) differed from day to day. In the L direction, the mean gauge length before testing on the second and third days increased to 70.37 mm and 79.53 mm, corresponding to strains of 75.9% and 98.8%, respectively. In the T direction, the corresponding mean gauge lengths were 63.41 mm and 68.67 mm, corresponding to strains of 58.52% and 71.65%, respectively.

**Figure 3:**
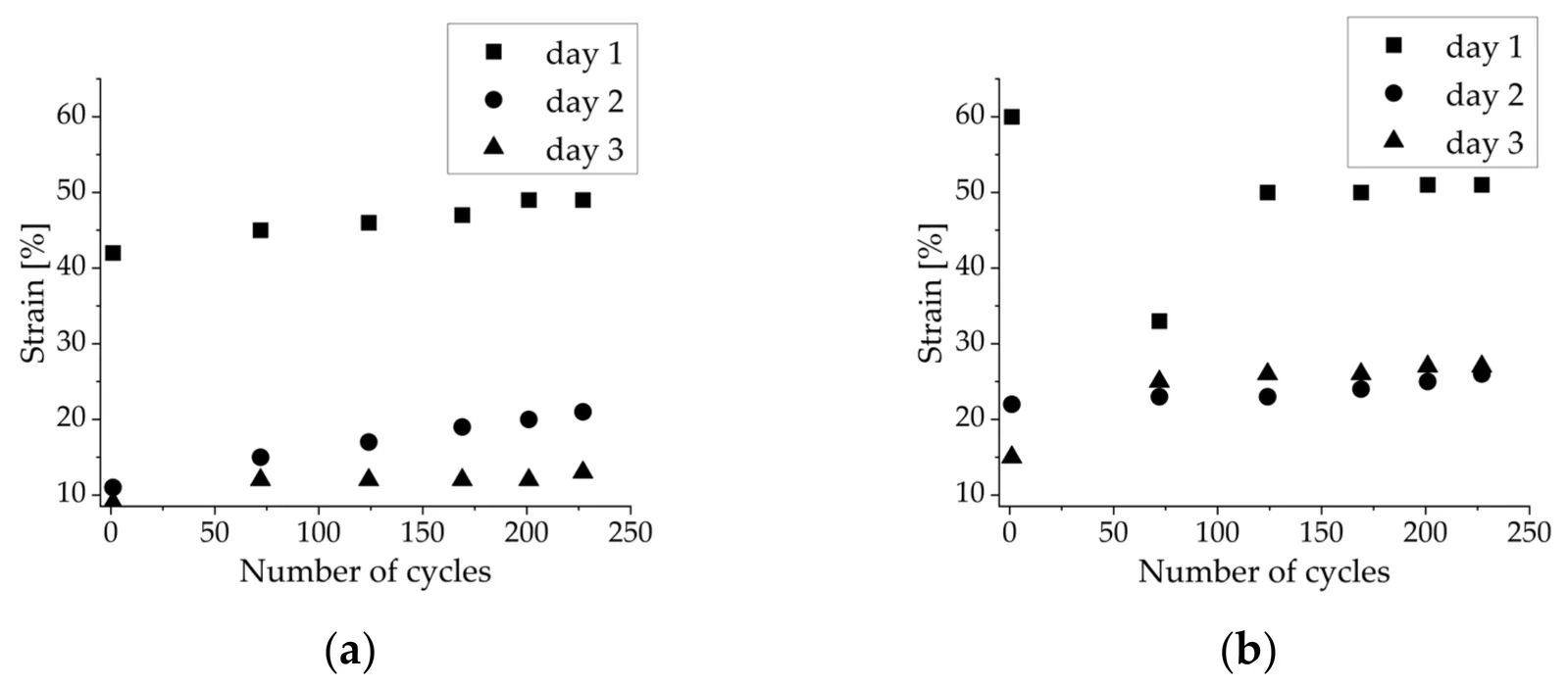
Evolution of strain of Specimen 1 during the cyclic loading protocol in the (**a**) L direction and (**b**) T direction.

The strain of the specimens after each simulated coughing bout was recorded (Figure 3). In the L direction, the specimens reached a strain of 43% of the initial gauge length during the first loading session at 09:00, which was significantly higher than the strains measured during all subsequent loading sessions. During the second and third testing days, the strain decreased markedly, reaching values of 11–22% and 9–14%, respectively. A similar trend was observed in the T direction. During the first loading session at 09:00, the specimens reached a strain of approximately 60%. During the loading episodes C2–C6 of the first testing day, the strain ranged from 33% to 53%. On the second and third testing days, the strain further decreased to 22–27% and 15–25%, respectively.

**Figure 4:**
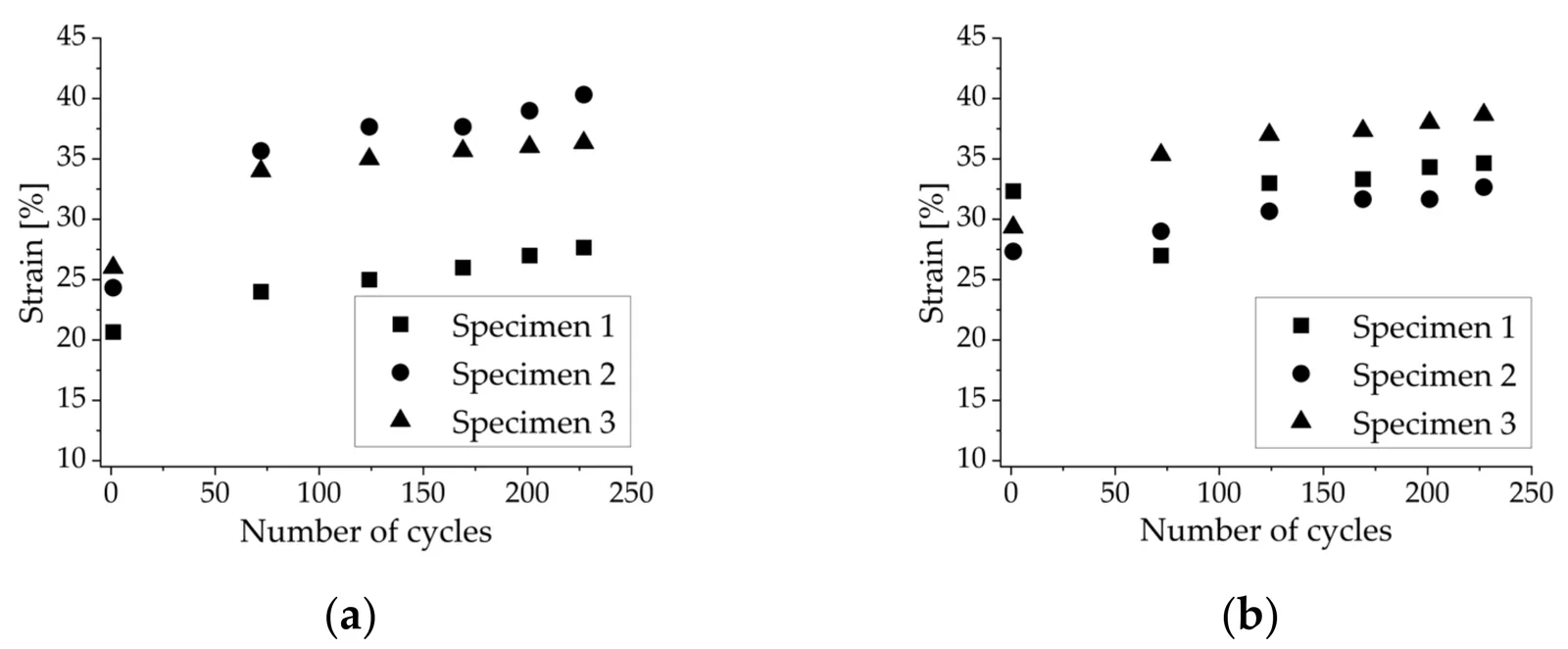
Mean strain at the end of loading scenario as a function of the cumulative number of loading cycles in the (**a**) L direction and (**b**) T direction.

The mean strain of the three specimens in the L and T directions calculated after ending the loading scenario is shown in Figure 4 as a function of the cumulative number of loading cycles. In both material directions, the mean strain increased progressively with the cumulative number of loading cycles, reflecting the accumulation of permanent deformation during repetitive loading. The mean strain increased by 5–15%, with the increase being more pronounced in the L direction than in the T direction.

The ability of the mesh material to recover during the resting periods between loading sessions was also investigated. The maximum strain recorded during each testing day decreased progressively after reaching peak values of 103% in the L direction at loading episode C1 and 83% in the T direction at C1 on the first day (after 71 loading cycles).

**Figure 5:**
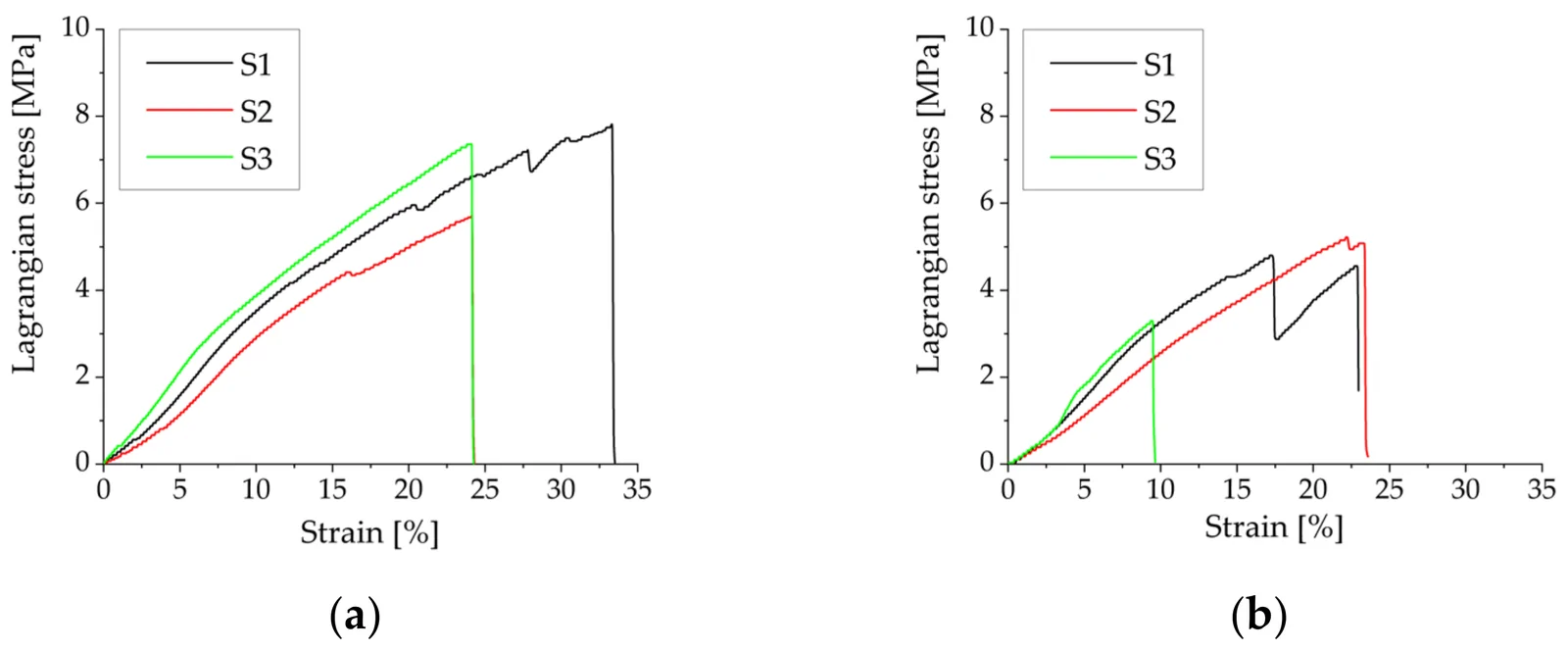
Stress–strain curves obtained from uniaxial tensile tests to failure after completion of the cyclic loading protocol in the (**a**) L direction and (**b**) T direction.

After completion of the cyclic loading protocol, the specimens were subjected to uniaxial tensile testing to failure. The failure stress ranged from 5.7 to 7.4 MPa in the L direction and from 3.3 to 5.3 MPa in the T direction (Figure 5).

**Figure 6:**
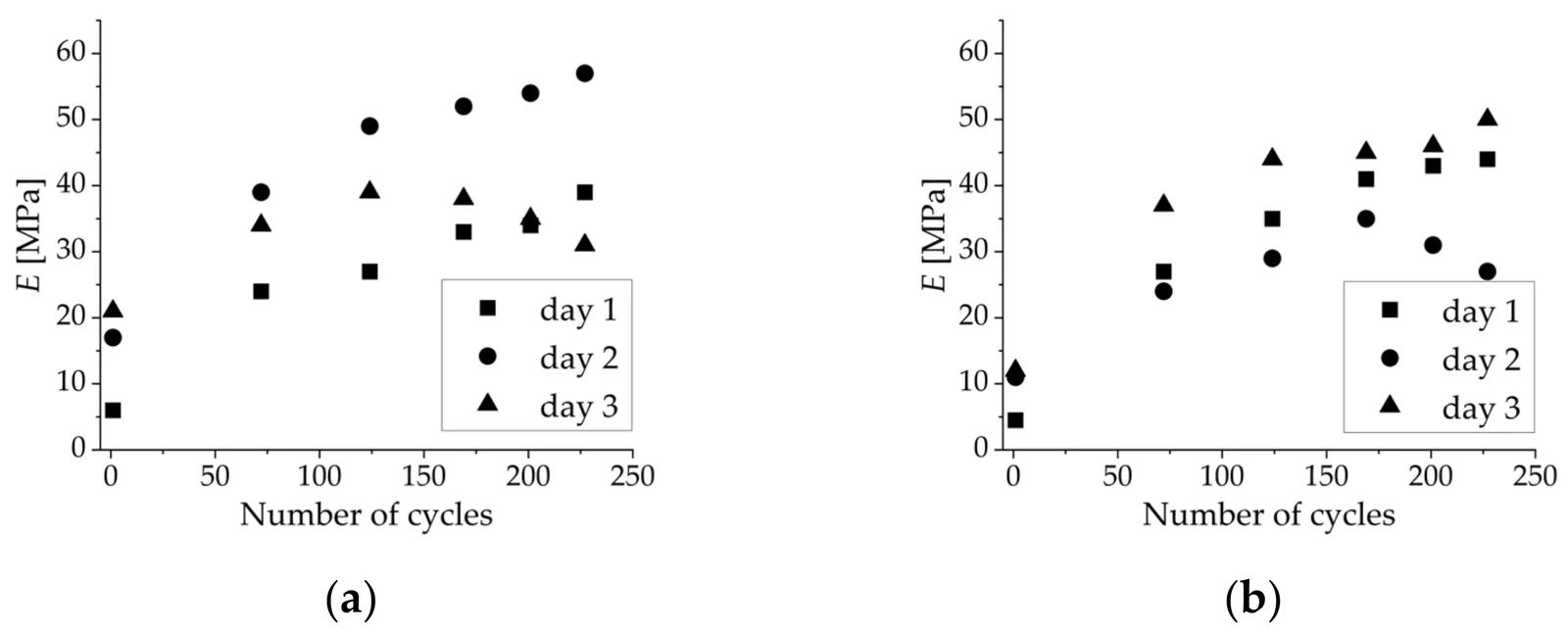
Elastic modulus *E* as a function of the cumulative number of loading cycles for Specimen 1 in the (**a**) L direction and (**b**) T direction.

Previous studies have shown that hernia meshes exhibit different stiffness during the initial loading cycle than during subsequent loading cycles within the physiological load range [19]. Consistent with these observations, the stiffness of the Microval specimens increased progressively with the number of loading cycles. The applied loading protocol resulted in at least a twofold increase in the elastic modulus within the physiological strain range. Figure 6 illustrates the evolution of the elastic modulus E of Specimen 1 as a function of the cumulative number of loading cycles during the three consecutive testing days in both the L and T directions.

**Figure 7:**
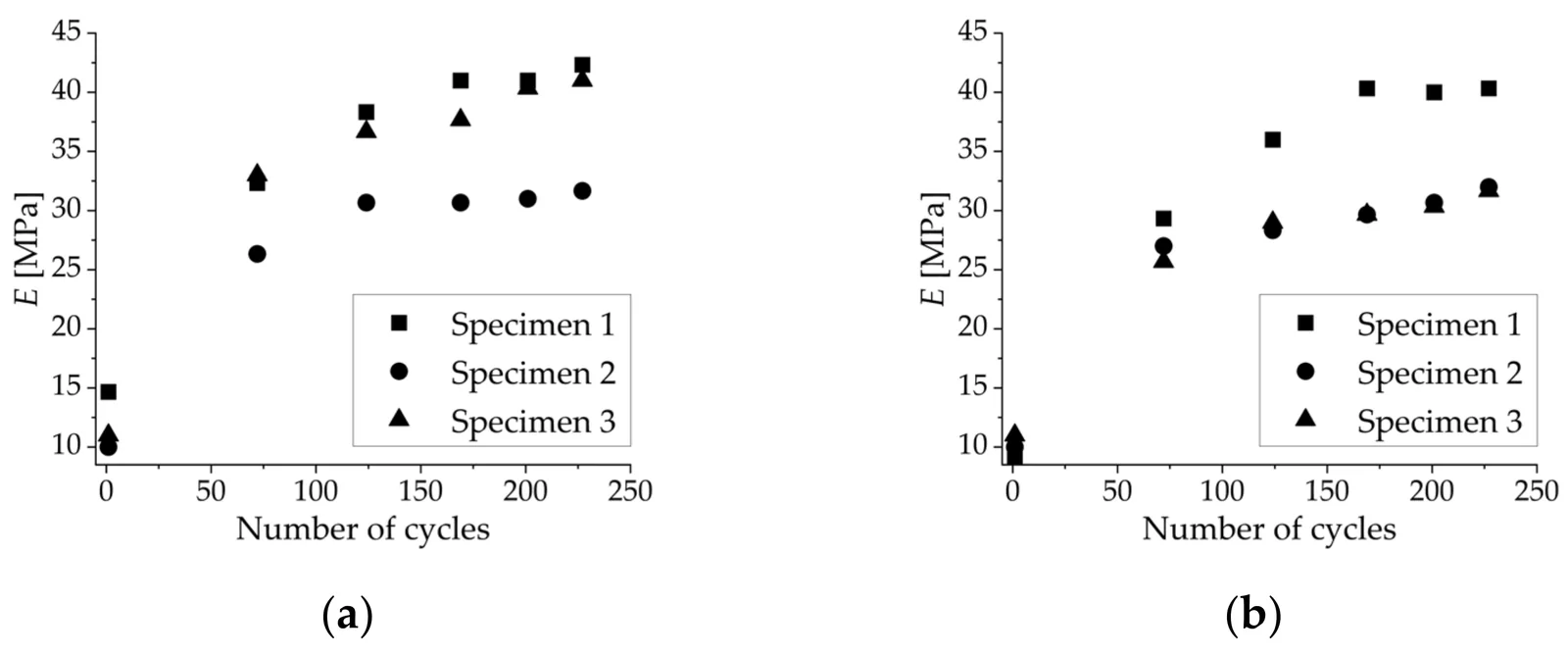
Mean elastic modulus *E* of the three tested specimens as a function of the cumulative number of loading cycles in the (**a**) L direction and (**b**) T direction.

The mean elastic modulus E of the three specimens as a function of the cumulative number of loading cycles is presented in Figure 7. The most pronounced increase in the elastic modulus occurred during the first 71 loading cycles.

**Figure 8:**
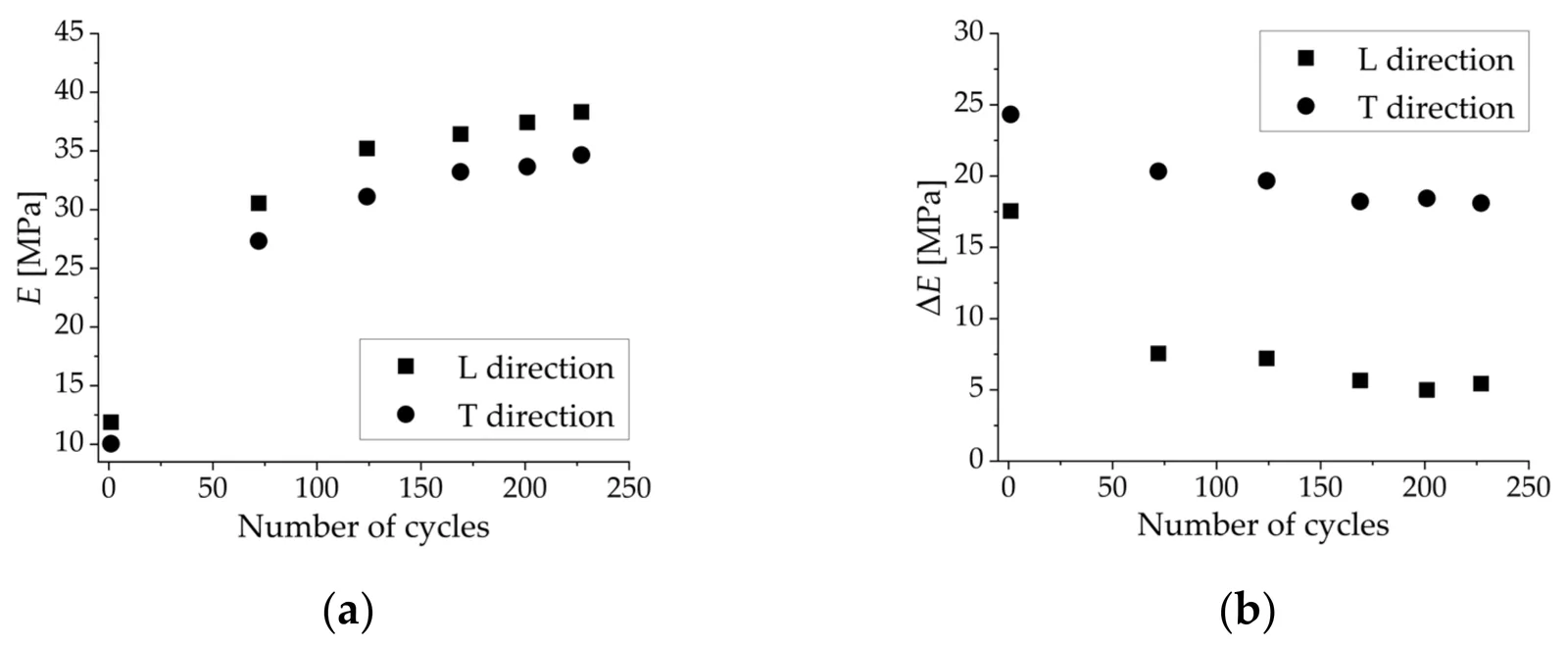
Mean elastic modulus *E* of the Microval mesh in the L and T directions (**a**). Change in elastic modulus Δ*E* between the initial state and after simulated coughing (**b**).

The mean elastic modulus E of the Microval mesh in the L and T directions is presented in Figure 8a. The elastic modulus increased approximately fourfold in both material directions during the cyclic loading protocol. No statistically significant difference was observed between the L and T directions (*p* = 0.53).

The change in elastic modulus (ΔE) after each simulated coughing bout was calculated relative to the initial elastic modulus and compared between the two material directions (Figure 8b). ΔE decreased approximately threefold in the L direction and 1.3-fold in the T direction. The difference between the two directions was statistically significant (*p* = 0.001).

The duration of each loading episode and the mean time per loading cycle were calculated. The mean duration of the loading episodes was slightly longer in the L direction (762. 74 ± 401.23 s) than in the T direction (601.36 ± 336.16 s) (Table 2). However, this difference was not statistically significant (*p* = 0.46).

The mean time per cycle (C1) required approximately 1.2 times longer to complete than the mean time per subsequent cycles C2–C6.

## 4. Discussion

Repetitive loading of hernia meshes occurs continuously in vivo as a result of fluctuations in intra-abdominal pressure (IAP). The highest physiological IAP values in healthy adults are generated during activities such as coughing and jumping. Consequently, dynamic mechanical testing is considered essential for evaluating mesh performance under breathing, coughing, sneezing, vomiting, and rising from a chair.

Despite the clinical importance of repetitive loading, relatively little information is available on the long-term mechanical response of hernia meshes subjected to cyclic loading. Most previous studies have focused on the early postoperative period, whereas the present study was designed to simulate a different clinical scenario. We assumed that several years after successful hernia repair, patients may develop chronic pulmonary diseases associated with persistent coughing, such as chronic obstructive pulmonary disease (COPD), asthma, or idiopathic pulmonary fibrosis (IPF). That is why the specimens prepared from aged Microval mesh that had exceeded its expiration date by 3 years were used, allowing evaluation of the mechanical performance of Microval under real loading conditions.

A uniaxial cyclic loading protocol was developed to reproduce repetitive loading associated with coughing. The applied loading conditions remained within the physiological range of subfailure loads and allowed assessment of the mechanical response of the mesh to repeated increases in IAP. This approach enabled evaluation of permanent deformation, changes in elastic modulus, and mechanical performance after prolonged cyclic loading.

One of the major challenges in developing a clinically relevant loading protocol is the large variability in cough frequency reported in the literature. Cough frequency depends strongly on the underlying disease, disease severity or time of day, and no consensus exists regarding the typical number of coughing episodes per hour in patients with chronic cough, COPD, asthma, or IPF. Several studies have reported substantially higher daytime than nighttime cough frequencies. For example, Key et al. reported a median 24 h cough frequency of 9.4 coughs per hour (range 1.5–39.4), with considerably higher daytime than nighttime rates (14.6 vs. 1.9 coughs per hour) [22].

Chung et al. monitored 97 patients with persistent cough over 30 days and demonstrated substantial inter- and intra-individual variability. Daily cough frequencies ranged from approximately 6 to more than 180 coughs per hour, highlighting the highly heterogeneous nature of chronic cough [23]. This variability supports the use of a loading protocol incorporating different numbers of loading cycles, as employed in the present study, to better represent the range of clinically relevant coughing patterns.

The studies of Siassi et al., Kallinowski et al., and Tomaszewska et al. demonstrated the importance of dynamic testing for evaluating hernia meshes under physiologically relevant loading conditions that simulate coughing, sneezing, and vomiting [19,24,25]. Siassi et al. developed a dynamic model for ventral hernia repair by applying internal pressures of 200 mmHg (≈27 kPa) to simulate coughing and 290 mmHg (≈39 kPa) to simulate vomiting [24]. Kallinowski et al. proposed a bench test based on low-amplitude cyclic loading and intermittent dynamic strain, demonstrating its potential for estimating the critical pressure resistance of hernia meshes before implantation [25]. Tomaszewska et al. investigated the mechanical response of the DynaMesh^®^-IPOM implant using tensile failure tests combined with cyclic loading–unloading experiments. They reported progressive stiffening of the material during cyclic loading and described the evolution of the elastic modulus using a rational-function approximation [19].

While these studies focused primarily on the immediate mechanical response of hernia meshes to cyclic loading, none reproduced a loading scenario incorporating repeated coughing episodes separated by physiologically relevant recovery periods. In the present study, the loading protocol included repeated daily loading sessions interspersed with short-term (25 min) and long-term (19 h) recovery intervals, thereby better reflecting the intermittent nature of chronic cough.

To the best of our knowledge, Krause et al. were the first to investigate the influence of aging on the mechanical behavior of non-implanted hernia meshes subjected to cyclic loading [17]. Nine commercial meshes stored under ambient laboratory conditions for 12 months were evaluated. Most materials exhibited reproducible mechanical behavior after aging, whereas Dexon and Prolene showed significant changes in mechanical properties. Cyclic loading produced permanent deformation ranging from approximately 8.5% to 19% strain [17].

Comparable observations were reported in our recent study on nine commercially available hernia meshes tested before and after their expiration date [18]. That study demonstrated that aging affects the mechanical characteristics of surgical meshes; however, the magnitude and direction of these changes depended on the mesh type.

The present study confirms the bilinear mechanical behavior previously reported for polypropylene meshes. The stress–strain curves exhibited pronounced hysteresis during cyclic loading in both directions, indicating the accumulation of permanent deformation. The progressive rightward shift of the hysteresis loops reflected the increase in the permanent extension of the specimens throughout the loading protocol. The mean gauge length increased to 75.9% and 98.8% above its initial value in the L direction after the second and third testing days, respectively, and to 58.5% and 71.7% in the T direction. The high strain values at the beginning of the loading scenario decrease to a range of 12% to 20% in the L direction and reach up to 25% in the T direction after completion of the three-day loading protocol (Figure 3).

These results indicate that repetitive loading associated with chronic coughing may progressively increase the permanent deformation of polypropylene meshes while reducing their ability to recover their original geometry between loading episodes.

The present study demonstrated progressive stiffening of the Microval mesh during uniaxial cyclic loading, as reflected by the increase in the elastic modulus. The mean elastic modulus increased approximately fourfold in both material directions throughout the loading protocol. However, the increase relative to the initial value Δ*E* was significantly greater in the L direction than in the T direction (*p* = 0.001).

The failure load (N) and the corresponding elongation at failure (mm) of samples from the described loading model were compared with the same parameters for the tested Microval mesh before the expiration date. Tensile tests were taken from [26]. After completion of the cyclic loading protocol, the specimens withstood maximum loads ranging from 32.0 to 43.7 N in the L direction and from 18.4 to 28.4 N in the T direction. The ultimate tensile stress of the cycled specimens remained within the same range (3.5–8.0 MPa) as that of the specimens from reference [26]. The elastic modulus and the elongation at failure were markedly altered following repetitive cyclic loading. These results indicate that repetitive cyclic loading primarily affected the deformability of the mesh, while its ultimate tensile strength remained largely unchanged.

Patterson et al. investigated the effect of cyclic loading on the mechanical performance of surgical meshes and reported that the instantaneous Young’s modulus increased rapidly during the first 15–30 loading cycles before reaching a plateau [14]. Similarly, Tomaszewska et al. observed that the elastic modulus of DynaMesh stabilized after approximately 20–40 loading cycles [16]. In contrast, the elastic modulus of the Microval mesh continued to increase throughout the entire loading protocol of 239 cycles (Figure 8), suggesting that a steady-state mechanical response had not yet been reached. Obviously, the stabilization of the elastic modulus would require a substantially greater number of loading cycles.

The clinical implications of cyclic loading have previously been discussed by Tomaszewska et al. and Jones et al. [13,16]. Jones et al. suggested that repeated increases in intra-abdominal pressure may produce progressive permanent deformation of implanted meshes, potentially contributing to postoperative complications [13]. Similarly, Tomaszewska et al. and Li et al. proposed that once a mesh undergoes permanent deformation, subsequent loading may further increase bulging of the abdominal wall [16,18]. The present findings support these observations by demonstrating the accumulation of residual strain and the progressive increase in elastic modulus under repetitive physiological loading conditions.

This study has several limitations. First, only one level of subfailure loading was investigated, although the extent of permanent deformation is expected to depend on the applied load. Future studies should therefore evaluate the mechanical response of hernia meshes under different physiological loading levels. Second, the number of loading cycles required to reach stabilization of the elastic modulus remains unknown and should be determined for Microval as well as for other commercially available meshes. The number of tested samples is insufficient for strong claims about the obtained results. The sample size should be increased for drawing strong statistical conclusions. Finally, the present investigation includes only expired Microval mesh. Additional studies involving different mesh types and varying periods after expiration are required to determine the general applicability of the observed mechanical behavior.

Overall, the results indicate that prolonged repetitive physiological loading primarily affects the deformability and stiffness evolution of the Microval mesh rather than its ultimate tensile strength, emphasizing the importance of cyclic mechanical testing when evaluating the performance of hernia meshes.

## 5. Conclusions

Understanding the mechanical behavior of hernia meshes under physiological loading is important for improving their mechanical evaluation and supporting implant selection. In this study, an in vitro cyclic loading protocol simulating repeated coughing episodes was developed to investigate the mechanical response of expired, non-implanted Microval mesh under a clinically motivated long-term loading scenario.

The results indicate that repetitive loading affected the deformability of the mesh more markedly than its ultimate tensile strength. Cyclic loading produced progressive permanent deformation and a substantial increase in the elastic modulus, whereas the ultimate tensile strength remained largely unchanged.

Unlike most previous studies, which focused on the early postoperative period, this work simulated repetitive coughing occurring several years after hernia repair using specimens prepared from Microval mesh beyond its expiration date. To the best of our knowledge, this is the first study reporting cyclic loading of non-implanted expired hernia mesh.

Although the present findings are specific to expired, non-implanted Microval mesh, they highlight the importance of cyclic mechanical testing for assessing the mechanical response of hernia meshes under repetitive physiological loading.

## Figures and Tables

**Table 1:** Hour-to-hour variability in cough counts for the monitoring period according to [21].

Time of Day	Loading Episode	Number of Cycles
9 a.m.	C1	71
10 a.m.	C2	52
11 a.m.	C3	45
12 a.m.	C4	32
1 p.m.	C5	26
2 p.m.	C6	13

**Table 2:** Duration of the loading episodes and mean time per loading cycle during the simulated coughing protocol in the L and T directions.

Loading Episode	Duration of the Loading Episode in the L Direction [sec]	Mean Time per Cycle in the L Direction [sec]	Duration of the Loading Episode in the T Direction [sec]	Mean Time per Cycle in the T Direction [sec]
C1	1498.55	21.10	1235.00	17.39
C2	964.22	18.54	752.88	14.45
C3	799.77	17.80	626.11	13.91
C4	605.88	18.93	446.55	13.95
C5	463.11	17.81	358.77	13.79
C6	244.88	18.83	188.88	14.53

## Data Availability

The datasets presented in this article are not readily available because the data are part of an ongoing study. Requests to access the datasets should be directed to Miglena Doneva.
